# Characterization of Incidental Liver Lesions: Comparison of Multidetector CT versus Gd-EOB-DTPA-Enhanced MR Imaging

**DOI:** 10.1371/journal.pone.0066141

**Published:** 2013-06-11

**Authors:** Yong Eun Chung, Myeong-Jin Kim, Yeo-Eun Kim, Mi-Suk Park, Jin Young Choi, Ki Whang Kim

**Affiliations:** Department of Radiology, Severance Hospital, Yonsei University College of Medicine, Seoul, Republic of Korea; University Hospital of Würzburg, Germany

## Abstract

As a result of recent developments in imaging modalities and wide spread routine medical checkups and screening, more incidental liver lesions are found frequently on US these days. When incidental liver lesions are found on US, physicians have to make a decision whether to just follow up or to undergo additional imaging studies for lesion characterization. In order to choose the next appropriate imaging modality, the diagnostic accuracy of each imaging study needs to be considered. Therefore, we tried to compare the accuracy of contrast-enhanced multidetector CT (MDCT) and Gd-EOB-DTPA-enhanced MRI for characterization of incidental liver masses. We included 127 incidentally found focal liver lesions (94 benign and 33 malignant) from 80 patients (M∶F = 45∶35) without primary extrahepatic malignancy or chronic liver disease. Two radiologists independently reviewed Gd-EOB-DTPA-enhanced MRI and MDCT. The proportion of confident interpretations for differentiation of benign and malignant lesions and for the specific diagnosis of diseases were compared. The proportion of confident interpretations for the differentiation of benign and malignant lesions was significantly higher with EOB-MRI(94.5%–97.6%) than with MDCT (74.0%–92.9%). In terms of specific diagnosis, sensitivity and accuracy were significantly higher with EOB-MRI than with MDCT for the diagnosis of focal nodular hyperplasia (FNH) and focal eosinophilic infiltration. The diagnoses of the remaining diseases were comparable between EOB-MRI and MDCT. Hence, our results suggested that Gd-EOB-MRI may provide a higher proportion of confident interpretations than MDCT, especially for the diagnosis of incidentally found FNH and focal eosinophilic infiltration.

## Introduction

Incidental lesions are defined as unexpected abnormalities found in patients without relevant symptoms [1]. As a result of recent developments in imaging modalities, more incidental liver lesions are found during medical examinations. According to previous reports, incidental liver lesions were found in 10% to 33% of imaging studies and more than 50% of autopsy cases [2], [3]. If patients have histories of prior malignancy or chronic liver disease, a differential diagnosis of metastasis or hepatocellular carcinoma (HCC) should be performed first. However, in the general population without prior relevant medical histories, the differential diagnosis should start at ground zero. As a result, incidentally found solid liver lesions are frequently difficult to characterize based on single imaging modalities and require further imaging or histological confirmation [2], [4]. According to a recent study, the overall diagnostic accuracy for incidentally found solid liver lesions was 52–66% when using gadolinium-based contrast-enhanced magnetic resonance (MR) imaging and 52–53% with contrast-enhanced ultrasound (CEUS) [5].

The recently introduced Gd-EOB-DTPA is a biphasic hepatobiliary MR contrast agent. Dynamic contrast-enhanced MR imaging can be performed with other Gd-based extracellular contrast agents and the hemodynamic or perfusion characteristics of the lesion can be assessed. Then, the hepatobiliary phase can be obtained as it undergoes both renal and biliary excretion. Observation of the hepatobiliary phase can provide histological and functional information about lesions and may improve the diagnostic accuracy of focal liver lesions [6]. According to previous reports, Gd-EOB-DTPA-enhanced MR can provide useful information not only for the detection of focal liver lesions, but also for the characterization of benign and malignant focal lesions in the liver. [7]. Despite the relatively large number of studies examining the detection and differential diagnosis of specific focal hepatic lesions on Gd-EOB-DTPA-enhanced MR, there have been no reports on the diagnostic performance of Gd-EOB-DTPA for the characterization of incidentally found focal liver lesions. Therefore, the purpose of our study was to assess the diagnostic performance of contrast-enhanced computed tomography (CT) and Gd-EOB-DTPA-enhanced MR for the differentiation of incidentally found solid liver lesions in patients without known histories of malignancy or chronic liver disease.

## Methods

### Patients

Our retrospective study was approved by the institutional review board of Yonsei University Health System, which issued a formal written waiver of informed consent. The inclusion criteria were as follows: [(1)] patients with incidentally found liver lesions on any of the imaging studies; and [(2)] patients who underwent 3- or 4-phase contrast-enhanced CT as well as Gd-EOB-DTPA-enhanced MRI. We excluded patients with prior histories of hepatic or extrahepatic malignancy and who regularly underwent follow-up evaluations because of chronic hepatitis. Simple hepatic cysts diagnosed using any of the imaging studies were also excluded during evaluation. Between June 2007 and June 2009, 178 MRIs were performed for the evaluation of incidental liver masses. Of 178 MRIs, 98 were excluded because of the following: 29 patients were lost to follow-up after imaging and a confirmative diagnosis was not made; 31 patients lacked 3- or 4-phase dynamic contrast-enhanced CT; and 38 patients had known histories of chronic liver disease, although they had no symptoms and had not undergone any imaging or laboratory screening. Finally, because there were no patients who had undergone more than 1 MRI, 80 patients (45 men, 35 women; mean age, 53.2 years; range, 29–85 years) with 127 incidentally found liver masses were included in our study.

The final diagnosis was made by US-guided biopsy (n = 2: 1 cholangiocarcinomas and 1 focal eosinophilic infiltration) and operation (n = 24: 1 AML, 3 cholangiocarcinomas, 1 embryonal sarcoma, 1 FNH, 13 HCCs, 2 hemangiomas, 2 inflammatory myofibroblastic tumor, and 1 schwannoma. The other 101 lesions were diagnosed by an experienced radiologist who did not participate in the imaging analysis based on the imaging findings including CT, MR, US, PET-CT and follow-up imaging of at least 1 year, clinical history and laboratory analysis including blood test, blood chemistry, and tumor markers. Among focal liver lesions, focal eosinophilic infiltration, AML, FNH, and HMG were regarded as benign lesions, whereas cholangiocarcinoma, embryonal sarcoma, and HCC were regarded as malignant lesions. Inflammatory myofibroblastic tumor was classified as a malignant lesion because it may present with locally aggressive behavior and can transform into a malignant tumor [8]. The median interval between CT and MR was 29 days (interquartile range [IQR], 13–79 days) for benign lesions and 6 days (IQR, 2–18 days) for malignant lesions.

### Ct and Mr Imaging Protocol

CT scans were performed with a 16- or 64-channel multidetector CT scanner (Somatom Sensation 16 and Sensation 64; Siemens Medical Solutions, Forchhein, Germany and Lightspeed VCT, GE Medical Systems, Milwaukee, WI, USA). First, a precontrast CT scan was performed before the administration of contrast media. Contrast media was injected by power injector via the antecubital vein in the amount of 2 mL/kg for 30 seconds. Using a bolus tracking technique, the late arterial phase was performed 18 seconds after the attenuation value reached 100 Hounsfield Unit (HU) at the abdominal aorta. The portal venous phase and delayed phase were obtained with a scan delay of 30 seconds and 150 seconds after the end of the previous phase.

MRI was performed with a 1.5-T or 3-T MR system (Achieva 1.5T, Philips Medical System, Best, The Netherlands; Tim Trio 3.0T, Siemens Medical Solutions, Forchhein, Germany) including a double echo T1-weighted gradient-echo image (in-phase/opposed-phased), respiratory triggered fat-saturated T2-weighted image, and 3-dimentional gradient echo T1-weighted image. Contrast-enhanced dynamic images were obtained at 25–35 sec (arterial phase), 55–65 sec (portal phase), 85–95 sec (equilibrium phase) and 10 min (hepatobiliary phase) after bolus injection of Gd-EOB-DTPA, 0.025 mmol/Kg body weight (Primovist, Bayer Schering Pharma, Berlin, Germany), followed by a saline flush of 15–20 mL, with the injection rate of 2 mL/sec.

### Image Analysis

Two radiologists (Y.E.K and Y.E.C with 6 (reviewer 1) and 3 (reviewer 2) years of experience in abdominal imaging) independently reviewed the CT and MR images in separate sessions. The interval between the two reading sessions was 1 month to avoid recall bias. All images were evaluated with a picture archiving and communication system (Centricity; GE Healthcare) without any patient information. In each session, reviewers were asked to evaluate whether the lesion was benign or malignant and to grade the confidence level on a six-point scale: 1, definitely benign; 2, probably benign; 3, possibly benign; 4, possibly malignant; 5, probably malignant; 6, definitely malignant. A diagnosis of a benign or malignant lesion was recorded as being correct when the reviewer diagnosed a benign lesion as benign with a confidence level of less than 2 or a malignant lesion as malignant with a confidence level of more than 5. Then, the reviewers were asked to provide the most appropriate diagnosis for each focal liver lesion. The confidence level of each specific diagnosis was graded on a five-point scale: 1, very unlikely; 2, unlikely; 3, likely; 4, very likely; 5, definitely [5]. A confidence level of 4 or 5 was regarded as being a correct diagnosis.

### Statistical Analysis

The diagnostic accuracy was calculated and compared between CT and MR with a generalized estimating equation method. Sensitivity, specificity and accuracy of specific diagnosis were calculated and compared between CT and MR by a generalized estimating equation method or weighted least squares method for repeated categorical data analysis. The agreement between CT and MR and interobserver variability between reviewers were calculated by weighted kappa statistics [9]. Statistical analysis was performed by a biostatistician using SAS (version 9.1.3, SAS Institute Inc., Cary, NC, USA). Differences with *P* values less than 0.05 were considered statistically significant.

## Results

### Diagnostic Performance

For the diagnostic accuracy of differentiation between benign and malignant lesions, CT showed an accuracy of 92.9% (95% confidence interval [CI]: 85.8–96.6) for reviewer 1 (R1) and 74.0% (95% CI: 61.7–83.5) for reviewer 2 (R2). With MR, the accuracy was 97.6% (95% CI: 92.8–99.3) for R1 and 94.5% (95% CI: 88.0–97.6) for R2. The diagnostic accuracy for the differentiation of benign and malignant lesions was significantly different between CT and MR for R2 (*P*<0.001), but not for R1 (*P* = 0.050), although the *P* value was marginal for the latter. Between reviewers, the diagnostic accuracy was significantly different for CT (*P*<0.001), but not for MR (*P* = 0.136).

In terms of diagnostic performance for specific types of lesions, MR showed greater sensitivity than CT for the diagnosis of FNH by R1 (P = 0.010) and focal eosinophilic infiltration by both reviewers (*P* = 0.020 for R1 and *P*<0.001 for R2) (**Table 1**). The accuracy of detection for specific diseases was significantly higher with FNH for both reviewers and focal eosinophilic infiltration for R2. Specificity was 100% for all four common lesions. For the diagnosis of relatively uncommon lesions, the sensitivities and specificities are summarized in **Table 2**.

**Table 1:** Sensitivity and accuracy of common lesions.

		Reviewer 1				Reviewer 2			
		Sensitivity	P value	Accuracy	P value	Sensitivity	P value	Accuracy	P value
Hemangioma (n = 45)	CT	86.7% (66.9–95.4)	0.058	95.3% (87.4–98.3)	0.063	75.6% (56.5–88.0)	0.054	91.3% (83.1–95.8)	0.068
	MR	97.8% (85.3–99.7)		99.2% (94.6–99.9)		86.7% (66.9–95.4)		95.3% (87.4–98.3)	
FNH (n = 27)	CT	66.7% (41.6–84.9)	0.010	92.9% (86.6–96.4)	0.017	37.0% (14.2–67.6)	0.060	86.6% (75.3–93.2)	0.045
	MR	96.3%(76.9–99.5)		99.2% (94.6–99.9)		77.8% (57.4–90.1)		95.3% (88.8–98.1)	
HCC (n = 24)	CT	95.8% (74.8–99.4)	0.231	99.2% (94.6–99.9)	0.236	79.2% (56.8–91.6)	0.716	96.1% (90.7–98.4)	0.715
	MR	83.3% (56.3–95.1)		96.9% (90.0–99.1)		83.3% (64.4–93.3)		96.9% (90.4–99.0)	
FEI* (n = 15)	CT	66.7% (31.0–89.9)	0.020	96.1% (89.3–98.6)	0.083	0%	<0.001	88.2% (76.3–94.6)	0.029
	MR	93.3% (62.4–99.2)		99.2% (94.6–99.9)		66.7% (5.3–96.6)		96.1% (89.3–98.6)	

Numbers in parentheses are the 95% confidence interval.

*FEI indicates focal eosinophilic infiltration.

**Table 2:** Sensitivity and accuracy of relatively uncommon lesions.

		Reviewer 1		Reviewer 2	
		Sensitivity	Specificity	Sensitivity	Specificity
Focal fat deposition	CT	2/3	124/124	1/3	124/124
	MR	2/3	124124	2/3	124/124
AML	CT	1/3	122/124	0/3	123/124
	MR	2/3	124/124	2/3	124/124
Schwannoma	CT	0/1	126/126	0/1	126/126
	MR	0/1	126/126	0/1	126/126
Cholangiocarcinoma	CT	6/6	121/121	3/6	121/121
	MR	6/6	117/121	6/6	121/121
Inflammatory myofibroblastic tumor	CT	0/2	125/125	0/2	125/125
	MR	0/2	125/125	0/2	125/125
Embryonal sarcoma	CT	0/1	126/126	0/1	126/126
	MR	0/1	126/126	0/1	126/126

### Inter- and Intra-Observer Variability

The weighted kappa value was 0.556 (95% CI: 0.478–0.634) for CT and 0.637 (95% CI: 0.568–0.706) for MR between R1 and R2. Between CT and MR, the weighted kappa value was 0.822 (95% CI: 0.746–0.897) for R1 and 0.609 (95% CI: 0.536–0.682) for R2.

### Descriptive Analysis of Uncertain and Missed Diagnoses

Of 55 hemangiomas, 1 lesion had an uncertain diagnosis (i.e., diagnosed as hemangioma with a confidence level of 3) from both reviewers (**Table 3**). This lesion showed peripheral dot-like enhancement, but central fill-in enhancement was not noted.

**Table 3:** Uncertain or misdiagnosed cases.

		Uncertain diagnosis		Misdiagnosis	
		CT	MR	CT	MR
Hemangioma	both	1	1	0	0
	R1	0	0	5	0
	R2	10	5	0	0
FNH	both	3	0	3	0
	R1	0	0	3	1
	R2	14	6	0	0
HCC	both	0	0	1	1
	R1	0	0	0	3
	R2	3	2	1	1
FEI	both	1	0	2	1
	R1	2	0	0	0
	R2	10	4	2	0

**Figure 1:**
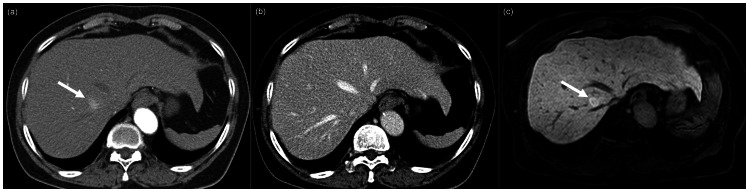
73-year-old female with incidental liver mass. (a) Arial phase CT image shows ill-defined hypervascular lesion in segment 8 of the liver (arrow). (b) The lesion shows isoattenuation on portal venous phase and both reviewers diagnosed this lesion as arterioportal shunt on CT with a confidence level of 4 and 3, respectively. (c) However, this lesion presented with high signal intensity compared to the adjacent normal liver on hepatobiliary phase of MR image (arrow). The diagnosis was changed to FNH on MR with a high confidence level of 5 by both reviewers.

Among 27 FNH, 3 lesions were misdiagnosed by both reviewers on CT. One lesion was misdiagnosed as AP shunt on CT by both reviewers, but diagnosed correctly as FNH on MR (**Figure 1**). Another lesion was misdiagnosed as AML by both reviewers on CT, but diagnosed as FNH on MR with a confidence level of 4 and 3. This lesion showed high signal intensity on HBP of MR. The last of the three misdiagnosed FHNs was diagnosed as hemangioma and HCC on CT by both reviewers, respectively. This lesion showed early heterogeneous enhancement in the arterial phase and persistent enhancement on the portal venous CT and MR images. On HBP of MR, the peripheral portion of the lesion showed high signal intensity, but the central portion showed low signal intensity compared with the adjacent liver parenchyma. This lesion was diagnosed as adenoma with a confidence level of 3 and FNH with a confidence level of 4 on MR by both reviewers.

**Figure 2:**
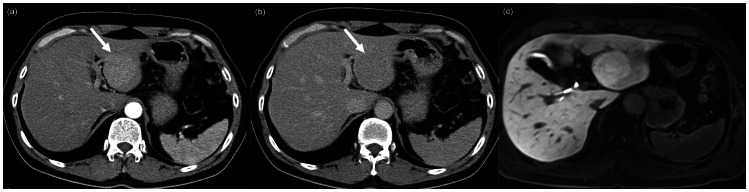
50-year-old man presented incidental liver mass. (a) On arterial phase CT image, faint arterial enhancing nodule is noted in the left lobe of the liver. (b) The lesion shows slightly low attenuation compared to adjacent liver on delayed CT image. Both reviewers diagnosed the lesion as HCC with a confidence level of 4 and 3 on CT. (c) On MR, the lesion showed high signal intensity on the hepatobiliary phase and the diagnosis was changed to FNH by both reviewers, but the lesion was confirmed as well-differentiated HCC by surgery.

Two HCC were misdiagnosed by both reviewers. One was misdiagnosed as FHN on CT by both reviewers because the lesion showed homogenous arterial enhancement without definite delayed washout. On MR, the lesion presented with low signal intensity compared with the adjacent liver on HBP and was diagnosed as HCC with a confidence level of 4 by both reviewers. The other HCC was diagnosed as HCC on CT by both reviewers with a confidence level of 4 (R1) and 3 (R2), but was misdiagnosed on MR as FNH with a confidence level of 5 (R1) and 3 (R2). This lesion showed high signal intensity on HBP and was confirmed as well-differentiated HCC (Edmonson grade I) by surgery (**Figure 2**).

Two focal eosinophilic infiltrations were misdiagnosed by both reviewers. One lesion was misdiagnosed as hemangioma by R1 (confidence level of 3) and cholangiocarcinoma by R2 (confidence level of 3). This lesion was diagnosed as focal eosinophilic infiltration with a confidence level of 4 on MR by both reviewers. The other lesion was misdiagnosed as peliosis by R1 (confidence level of 3) and cholangiocarcinoma by R2 (confidence level of 3) both on CT and MR.

## Discussion

Our results demonstrate that Gd-EOB-DTPA-enhanced MR provides better diagnostic accuracy for the differentiation of benign and malignant lesions and for the specific diagnosis of FNH and focal eosinophilic infiltration. Both dynamic contrast-enhanced CT and Gd-EOB-DTPA-enhanced MR had similarly high diagnostic performances for hemangioma and HCC, whereas relatively uncommon lesions such as inflammatory myofibroblastic tumor, schwannoma or embryonal sarcoma were rarely diagnosed accurately on both CT and MR.

According to a previous study, about 37% of patients with incidental lesions that were found on whole body CT scans during medical checkups received at least one recommendation for further evaluation [4]. Several guidelines have been suggested for radiologists or clinicians when they encounter incidental liver lesions [1], [2]. But these guidelines cannot be applied uniformly because disease epidemiology varies according to geographic area and medical history or conditions vary between patients. When incidental liver lesions are found, the main role of the physician is either to make an appropriate diagnosis without performing unnecessary procedures or to recommend the next appropriate diagnostic step. In our results, Gd-EOB-DTPA MR showed a similar diagnostic performance to contrast-enhanced MDCT for most incidental liver lesions, although statistical significance was only noted in the diagnosis of FHN and focal eosinophilic infiltration. Furthermore, radiation exposure is not needed during MR, hence patients who are vulnerable to radiation exposure, such as children, young females or pregnant women, MR is a more appropriate imaging study for the diagnosis of incidental liver lesions. However these results do not suggest that MR is always superior to MDCT for characterization of incidental liver lesions. This is because CT evaluates not only the liver but also other abdominal solid organs, hollow viscous, peritoneal cavities and even basal lungs in one examination. Examination time is also much shorter in CT than in MR, which is important to patients who have claustrophobia. Hence CT and MR should be chosen case by case, based on clinical situations.

In our study, inter-observer agreement between CT and MR was higher for the more experienced radiologist than for the less experienced radiologist, and intra-observer agreement between CT and MR was higher for the more experienced radiologist than for the less experienced radiologist. These results suggest that more experienced radiologists could diagnose incidentally found liver masses both on CT and MR, whereas less experienced radiologists might diagnose incidental liver lesions more accurately and confidently on MR than on CT.

According to a previous study, CEUS could correctly differentiate benign from malignant lesions in about 96.5% of cases and yield a correct specific diagnosis in 52%–73% of cases [5], [10]. It was also reported that CEUS showed comparable diagnostic performance with Gd-chelate contrast-enhanced MR for incidental liver lesions [5]. CEUS also has merits in that it does not involve radiation exposure, requires a shorter exam time than MR, and is relatively less expensive than CT and MR. But CEUS, like conventional gray-scale US, is operator-dependent, requires a dedicated US sequence and is limited in some regions because the US contrast agent is still not commercially available. Furthermore, the diagnostic performance of CEUS in the previous study might be overestimated because less than 10% of the included incidental liver lesions were malignant and most of the malignant lesions were diagnosed with uncertainty [5], [10].

Hepatic hemangioma could be diagnosed accurately both on CT and MR because it usually presents with typical imaging findings of peripheral globular enhancement with gradual central fill in [2]. However, slow enhancing hemangioma might present as a low attenuating lesion even in the delayed phase and could be diagnosed as hypovascular metastasis or cystic lesion on CT [11]. Early and persistent enhancing hemangioma could be misdiagnosed as a hypervascular tumor such as HCC or neuroendocrine carcinoma on CT and even on MR because hemangioma might manifest as a relatively low SI compared to the adjacent normal liver parenchyma, which uptakes Gd-EOB-DTPA during the equilibrium phase, i.e., pseudo-washout sign [12]. Findings with high signal intensity on T2-weighted MR image could be helpful for diagnoses of hemangioma with atypical enhancement patterns [11]. According to previous studies, MR showed better accuracy for the diagnosis of hemangioma than CT (84% for MR versus 73% for CT) [13]. Our result was compatible with that of a previous study, where the sensitivity and accuracy were higher on MR (sensitivity: 86.7–97.8%, accuracy: 95.3%–99.2%) than on CT (sensitivity: 75.6%–86.7%, accuracy: 91.3%–95.3%), although there was no statistically significant difference.

According to previous reports, Gd-EOB-DTPA-enhanced MRI (88.1%) showed superior diagnostic performance in comparison with contrast-enhanced CT (84.7%) or non-enhanced MR (67.8%) for the diagnosis of FNH [14]. FNH usually presents with hypervascular nodules on the arterial phase that return to iso-attenuation or iso-signal intensity on portal venous or equilibrium phase images [14]. However, this enhancement pattern is not sufficient for the diagnosis of FNH, especially in small lesions, because other lesions such as HCC, hypervascular metastasis, and adenoma could be manifested in this way. Tortuous feeding vessels and central scars might be helpful for the diagnosis and MR could show central scars more clearly because of its high tissue contrast. Furthermore, the hepatobiliary phase on Gd-EOB-DTPA-enhanced MR could give additive information since FNH usually presents with high- or iso-SI compared with the adjacent liver on the hepatobiliary phase [14], [15]. As a result, MR may have had a higher sensitivity and accuracy than CT for the diagnosis of FNH in our study.

Gd-EOB-DTPA-enhanced MR showed comparable or better diagnostic performance for the diagnosis of HCC than contrast-enhanced CT, gadopentetate dimeglumine-enhanced MR imaging [16], [17]. The study samples of previous studies included patients with chronic liver disease or liver cirrhosis. If HCC develops in the cirrhotic liver and information such as serum alpha fetoprotein level and clinical history are given to the radiologist, HCC could be easily diagnosed. Incidentally found HCC is often diagnosed correctly or might be diagnosed as HCC with a lower confidence level because it frequently presents as a large dominant mass and is accompanied by calcification, hemorrhage, a fat component, dilated intrahepatic bile duct, and abdominal lymphadenopathy, which are not common findings for typical HCC that develops in the cirrhotic liver [18]. Furthermore, a hypervascular nodule on arterial phase images with a high signal intensity on hepatobiliary phase MR image might easily be misdiagnosed as FNH, especially in patients with non-cirrhotic liver. In our study, one HCC was diagnosed correctly as HCC on CT, but misdiagnosed as FNH on MR by both reviewers because the lesion showed high signal intensity on the hepatobiliary phase. In this case, a hepatobiliary phase MR image would actually interfere with the diagnosis.

In terms of focal eosinophilic infiltration, it has been reported that portal phase CT image shows better lesion-to-liver contrast than that of Gd-chelate contrast-enhanced MR image [19]. However, recently used Gd-EOB-DTPA-enhanced MR offers better lesion-to-liver contrast on portal venous or equilibrium phase MR images than CT or Gd-chelate-enhanced MR images because about 50% of the contrast medium is taken up by hepatocytes, resulting in increased signal intensity of the normal liver parenchyma [20]. In our study, the sensitivity for diagnosis of focal eosinophilic infiltration was only 0%–66.7% on CT. However, most of the diagnoses were not misdiagnoses, but rather, diagnoses with a lower confidence level (i.e. confidence level less than 3). This might be because the reviewers were blinded to information other than CT images, such as peripheral eosinophil count, which is very informative for the diagnosis of focal eosinophilic infiltration [20]. On MR, the sensitivity increased to 66.7%–93.3%. Although metastasis cannot always be differentiated from focal eosinophilic infiltration, enhancement patterns and the margin of the lesion, which is homogenous or shows rim enhancement on arterial phase, and low-attenuation or low-signal intensity on the delayed- and hepatobiliary-phase with ill-defined margin could be helpful findings for differential diagnosis and these findings could be more clearly depicted on Gd-EOG-DTPA-enhanced MR than CT [20].

For the less common lesions, especially inflammatory myofibroblastic tumor, schwannoma and embryonal sarcoma, none were diagnosed correctly by both reviewers. Because of the rarity of these lesions, it is hard to accurately diagnose them in almost all cases, although they might be included in the differential diagnosis. In contrast, focal fat deposition and cholangiocarcinoma could be diagnosed properly on CT or MR because of their specific locations or typical imaging findings. In terms of AML, if it presents with a gross fat component, it can be diagnosed directly as AML or as one of the differential diagnoses including fat-containing liver tumors such as lipomas or HCC with fatty metamorphosis.

There are several limitations of our study. First, this study was retrospective. Second, we evaluated incidental liver lesions based only on imaging findings, although clinical findings including physical examination, laboratory test, age and gender are also important and helpful for differential diagnosis. Third, we excluded patients with prior histories of malignancy or chronic hepatitis. In these patients, it might be more difficult to differentiate metastasis or HCC from other benign or malignant lesions.

In conclusion, for the characterization of incidental liver masses, Gd-EOB-DTPA-enhanced MR provides better diagnostic accuracy than CT for the differentiation of benign and malignant lesions and for the specific diagnosis of FNH and focal eosinophilic infiltration.
