# Correction: Characterization of Incidental Liver Lesions: Comparison of Multidetector CT versus Gd-EOB-DTPA-Enhanced MR Imaging

**DOI:** 10.1371/annotation/a03efa25-49f8-4616-a256-074a3d60ceaf

**Published:** 2013-10-24

**Authors:** Yong Eun Chung, Myeong-Jin Kim, Yeo-Eun Kim, Mi-Suk Park, Jin Young Choi, Ki Whang Kim

The last author was incorrectly indicated as the Corresponding Author for the article; the Corresponding Author is the second author, Myeong-Jin Kim. The email address currently indicated as being for the last author is the second author's correct email address. 

